# Thymic Neuroendocrine Tumors: Evolving Insights and Innovative Approaches

**DOI:** 10.1016/j.jtocrr.2025.100935

**Published:** 2025-11-20

**Authors:** Erica Pietroluongo, Christine M. Bestvina, Rachel Brattin, Pietro De Placido, Anna Di Lello, Waqas Haque, Alessandra Esposito, Roberto Bianco, Noura Choudhury, Marina Chiara Garassino

**Affiliations:** a Department of Medicine, Section of Hematology Oncology, Thoracic Oncology Program, The University of Chicago Medicine, Chicago, Illinois; b Department of Clinical Medicine and Surgery, University Federico II, Naples, Italy; c Department of Advanced Biomedical Sciences, University of Naples Federico II, Naples, Italy; d Department of Medical Oncology, Dana-Farber Cancer Institute, Boston, Massachusetts; Harvard Medical School, Boston, Massachusetts; e Medical Oncology Department, IRCCS San Raffaele Hospital, Milan, Italy

**Keywords:** Thymic neuroendocrine tumors, Targeted therapy, PRRT, Somatostatin analogs

## Abstract

**Introduction:**

Thymic neuroendocrine tumors (TNENs) are exceptionally rare and a clinically heterogeneous malignancy, often diagnosed at an advanced stage and lacking standardized treatment algorithms. Due to the scarcity of dedicated evidence, most therapeutic strategies are extrapolated from other neuroendocrine neoplasms.

**Methods:**

This narrative review provides an updated overview of current and emerging treatment approaches for TNENs, focusing on histology-driven strategies and the evolving role of targeted and radionuclide therapies. A comprehensive literature search was conducted through PubMed/MEDLINE and Embase from January 1, 2000, up to May 31, 2025, integrating retrospective series, real-world data, and ongoing clinical trials.

**Results:**

Surgical resection remains the cornerstone of treatment whenever feasible. The benefit of adjuvant therapy in well-differentiated tumors is unclear, whereas thymic neuroendocrine carcinomas often require multimodal approaches, including platinum–etoposide chemotherapy and radiotherapy. Retrospective evidence suggests that even well-differentiated, high-grade tumors may respond to cytotoxic agents. Somatostatin analogues are widely used in indolent or peptide receptor-positive tumors, whereas everolimus and, more recently, cabozantinib represent options for progressive disease. Peptide receptor radionuclide therapy has demonstrated encouraging results in somatostatin receptor–positive tumors and is currently under further investigation in prospective trials involving thymic primaries. However, the 5-year overall survival rate varies significantly (approximately 28%–80%), underlining an urgent need for prospective, subtype-specific studies.

**Conclusions:**

The management of TNENs requires a multidisciplinary and individualized approach based on histologic subtype, somatostatin receptor status, and disease aggressiveness. Despite promising therapeutic options, robust prospective data remain limited. The integration of TNENs into basket trials, the molecular refinement of prognostic subgroups (e.g., NET G3), and the conduct of dedicated multicenter prospective studies are urgently needed to define optimal treatment algorithms and improve clinical outcomes in these rare entities.

## Introduction

Thymic neuroendocrine tumors (TNENs) originate from neuroendocrine cells within the thymus and represent a rare and heterogeneous group of tumors. They account for less than 5% of all thymic malignancies and less than 1% of neuroendocrine tumors (NENs) when excluding pulmonary carcinoids. The age-adjusted incidence rate ranges from 0.02 to 0.18 per 100,000 inhabitants annually in Europe or the United States, respectively.^1^^,^^2^

These tumors comprise a spectrum of neoplasms with varying behaviors, ranging from well-differentiated typical and atypical carcinoids (collectively TNETs) to highly aggressive, poorly differentiated large-cell neuroendocrine carcinomas (LCNECs) and small-cell neuroendocrine carcinomas (SCNECs), collectively TNECs.^3^ Compared with thymomas, TNENs are considered to be more aggressive, with a heterogeneous 5-year overall survival (OS) rate ranging from 28% to 72%.^4^ A recent large retrospective cohort of advanced TNETs reported a median OS of 110 months and a 5-year survival rate above 80%, indicating biological heterogeneity and underscoring the limitations of Ki-67 and WHO grading in prognostic assessment.^4^

TNENs demonstrate a slight male predominance and typically present in the fifth to sixth decades of life, with atypical carcinoids more common among the White population. In sporadic cases, ectopic Cushing syndrome is reported in 15% to 20% of patients and is associated with poor prognosis. In contrast, TNENs arising in the context of multiple endocrine neoplasia type 1 (MEN-1) are predominantly atypical carcinoids and are usually nonfunctioning.^1^, ^2^, ^3^, ^4^ The rarity and diverse biological behaviors within TNENs pose significant diagnostic and treatment challenges. This narrative review aims to provide an updated overview of TNENs, focusing on histopathologic classification and current and emerging therapeutic strategies.

## Methods

A comprehensive narrative review was conducted to synthesize current evidence on TNENs, spanning from well-differentiated carcinoids (TNETs) to poorly differentiated neuroendocrine carcinomas (TNECs). The literature search was performed in PubMed/MEDLINE and Embase databases up to May 31, 2025. The search strategy combined MeSH/Emtree terms and free-text keywords, including the following: “thymic neuroendocrine tumor,” “thymic carcinoid,” “atypical carcinoid of thymus,” “thymic large cell neuroendocrine carcinoma,” “thymic small cell carcinoma,” and “NET G3 thymus.” Only articles published in English and involving research on human subjects were considered. Additional relevant data were retrieved through manual screening of reference lists and proceedings from major oncology and NEN conferences (e.g., ASCO, ESMO, ENETS, ITMIG), to capture the most updated clinical, pathologic, and therapeutic evidence.

## Classification

The WHO 2021 classification system maintained the previous one, dividing TNENs into the following four main categories based on their histopathologic features: typical carcinoid (TC), atypical carcinoid (AC), small cell neuroendocrine carcinoma (SCNEC), and large cell neuroendocrine carcinoma (LCNEC). Each category has distinct characteristics that influence prognosis and treatment strategies (Table 1).^3^

• Typical carcinoids are well-differentiated tumors with low mitotic rates (<2 mitoses per 2 mm^2^) and no necrosis. These tumors display a uniform population of cells with round nuclei, fine chromatin, and moderate cytoplasm. Typical carcinoids express neuroendocrine markers, including chromogranin A, synaptophysin, and CD56, as determined by immunohistochemistry (IHC).^1^^,^^3^
• Atypical carcinoids are well-differentiated NENs with a higher mitotic rate (2–10 mitoses per 2 mm^2^) and focal necrosis. These neoplasms exhibit more significant cellular atypia and architectural disorganization than typical carcinoids. IHC staining is analogous to that observed in typical carcinoids, exhibiting positive expression of neuroendocrine markers.^1^^,^^3^
• SCNECs are highly aggressive, poorly differentiated NENs with small, round to oval cells, scant cytoplasm, and a high mitotic rate. These tumors are frequently accompanied by extensive necrosis and a high Ki-67 proliferation index. IHC analysis reveals that SCNECs are positive for neuroendocrine markers and may also express cytokeratin.^1^^,^^3^
• LCNECs are poorly differentiated NETs characterized by large cells with abundant cytoplasm, prominent nucleoli, and high mitotic activity (>10 mitoses per 2 mm^2^). These tumors frequently exhibit extensive necrosis and a high Ki-67 proliferation index. LCNECs express neuroendocrine markers but may also demonstrate variable expression of epithelial markers, such as cytokeratin.^1^^,^^3^

**Table 1:** Histopathologic and Clinical Features of Thymic Neuroendocrine Tumors

Subtype	Differentiation	Mitoses/Ki-67^a^	Clinical Behavior	SSTR Expression	PRRT Eligible
Typical carcinoid	Well differentiated	<2 mitoses/2 mm^2^ Ki-67 usually <5%	Indolent but often diagnosed at advanced stage	Frequently positive	Yes (if SSTR+)
Atypical carcinoid	Well differentiated	2–10 mitoses/2 mm^2^ Ki-67 5%–20%	Intermediate aggressiveness	Often positive	Yes (if SSTR+)
LCNEC	Poorly differentiated	>10 mitoses/2 mm^2^ Ki-67 usually >40%	Aggressive, often metastatic at diagnosis	Rarely positive	Rare
SCNEC	Poorly differentiated	>10 mitoses/2 mm^2^ Ki-67 often >60%	Highly aggressive, early systemic spread	Rarely positive	Rare

*Note:* Illustrates differentiation grade, proliferative indices, clinical behavior, somatostatin receptor (SSTR) expression, and potential eligibility for PRRT across thymic neuroendocrine tumor subtypes.

LCNEC, large-cell neuroendocrine carcinoma; PRRT, peptide-receptor radionuclide therapy; SCNEC, small-cell neuroendocrine carcinoma; TNEN, thymic neuroendocrine tumor.

a Although the Ki-67 proliferation index does not constitute a formal criterion for the grading of TNENs in the WHO 2021 classification, it is nonetheless included in this table to reflect its widespread use in clinical and research settings as a marker of biological behavior. Incorporating Ki-67 values facilitates the characterization of tumor subtypes, particularly in distinguishing well-differentiated neoplasms with elevated proliferative activity, often referred to as NET G3, from poorly differentiated neuroendocrine carcinomas and may have relevant implications for prognostication and therapeutic decision-making.

In addition, a subset of TNETs called “NET G3,” not formally included in the current WHO classification, has been identified based on analogies with gastro-enteropancreatic (GEP) and pulmonary neuroendocrine classifications. These tumors retain well-differentiated morphology despite displaying high proliferative activity. Specific IHC features may help distinguish NET G3 from true LCNEC.^3^ A “NET G3” subgroup often demonstrates an IHC pattern of chromogranin A positivity, EZH2 negativity, SSTR2A expression, and retained RB/TP53, contrasting with true LCNEC, which more frequently exhibits EZH2 overexpression, RB loss, and abnormal p53. EZH2 levels correlate with poorer outcomes and may serve as a potential therapeutic target. Recurrent NF1 alterations, especially in NET G3/LCNEC, suggest a potential RAS–MAPK pathway dependence.^5^

From a molecular perspective, TNETs exhibit diverse but organized genomic patterns. Integrative profiling identifies three copy-number instability (CNI) levels, CNI_low at less than 9, CNI_intermediate at 9 to less than 30, and CNI_high at more than or equal to 30, which provide prognostic information and are not reliably predicted by morphology or Ki-67. The somatic copy-number load generally increases from typical to atypical carcinoid and is highest in LCNEC/SCNEC, whereas 11q deletions, common in pulmonary carcinoids, are rare in TNET. Long-term observations indicate that biological evolution may involve both a histologic “grade shift” and increasing CNI over time.^5^

However, the molecular pathways driving and differentiating low-grade (TC and AC) and high-grade TNENs (SCNEC and LCNEC) are not fully understood, nor is it known whether these pathways differ from those in pulmonary tumors.

Notably, unlike in GEP and pulmonary NENs, the Ki-67 index is not currently incorporated into the grading of TNENs, creating uncertainty regarding the optimal management of well-differentiated tumors with high Ki-67 values.

Supporting this concept, a comprehensive, multi-network retrospective study re-evaluated 74 cases of advanced TNETs (excluding NECs) through centralized pathologic review and observed that 31% of cases displayed well-differentiated morphology combined with a high Ki-67 index, suggesting features consistent with NET G3. However, formal diagnostic criteria for this category are not yet established in thymic epithelial tumors (TETs).^3^^,^^5^^,^^6^ This underscores the biological heterogeneity of TNETs and raises critical questions regarding the prognostic relevance and therapeutic approach for this emerging subgroup.

## Clinical Presentation

TNENs usually present with nonspecific thoracic symptoms, such as cough, chest pain, dyspnea, or superior vena cava syndrome, which typically reflect the local mass effect of mediastinal disease. Paraneoplastic and autoimmune manifestations occur in approximately 20% to 25% and may occur because of the production of abnormal hormones or immune-mediated mechanisms triggered by the tumor, which can make the clinical presentation and management complicated, requiring a multidisciplinary treatment approach.^7^ Among paraneoplastic complications, endocrine syndromes, though relatively uncommon, are clinically relevant because they can dominate the clinical features and significantly affect prognosis and management.^8^ In addition, immune-mediated manifestations, including autoimmune cytopenias and neuromuscular disorders, may further complicate the diagnostic process. Given this heterogeneity, systematic biochemical evaluation and multidisciplinary management are recommended.

### Cushing’s Syndrome

Cushing’s syndrome is the most common hormone-related complication, affecting approximately 15% to 20% of sporadic TNENs and usually linked to a poor prognosis.^9^ It results from ectopic adrenocorticotropic hormone (ACTH) secretion by tumor cells. Clinical features include central obesity, moon facies, hypertension, hyperglycemia or overt diabetes, proximal muscle weakness, osteoporosis, thinning skin with violaceous striae, and mood changes.^10^ Diagnosis is confirmed by elevated serum cortisol and ACTH, 24-hour urinary free cortisol, and dexamethasone suppression test results, with localization aided by somatostatin-receptor positron emission tomography (PET)/computed tomography (CT).^11^ Complete surgical removal is the preferred treatment when possible; medical options such as steroidogenesis inhibitors (e.g., ketoconazole, metyrapone) or bilateral adrenalectomy may be used in refractory cases.^12^

### Carcinoid Syndrome

Carcinoid syndrome is rare (<5%) and far less common in TNENs than in pulmonary or gastroenteropancreatic neuroendocrine tumours, and it is virtually absent in TNECs.^13^^,^^14^ It results from systemic release of serotonin, tachykinins, and prostaglandins, leading to episodic flushing, diarrhea, wheezing, abdominal cramps, and, in longstanding cases, carcinoid heart disease. Biochemical confirmation rests on 24-hour urinary 5-hydroxyindoleacetic acid (5-HIAA) (dietary preparation recommended) and plasma serotonin.^15^ Management includes somatostatin analogues for symptom control,^16^ cytoreductive surgery when feasible,^17^ and peptide-receptor radionuclide therapy (PRRT) for advanced disease; telotristat ethyl may be used in cases of refractory diarrhea.^18^

### Other Rare Syndromes

Other hormone-mediated syndromes are exceedingly rare (<3%) but may have important clinical implications. These include acromegaly due to ectopic growth hormone–releasing hormone secretion,^19^ hypertrophic osteoarthropathy,^20^ syndrome of inappropriate antidiuretic hormone secretion (SIADH),^21^ and parathyroid hormone-related peptide (PTHrP)–mediated hypercalcemia.^22^ Although anecdotal, their recognition is important, as timely diagnosis and targeted treatment may modify management and improve outcomes.

In addition, TNENs may manifest in the spectrum of MEN-1, occurring in nearly 20% to 25% of patients with the syndrome. MEN-1, also known as Warmer syndrome, is a hereditary disorder caused by mutations in the MEN1 gene that leads to several endocrine and non-endocrine tumors. TNENs represent a major cause of mortality in MEN-1 patients, along with pancreatic NENs and parathyroid carcinoma, due to their aggressive behavior, late presentation, and high metastatic potential. TNENs in MEN-1 typically present at an advanced stage, which complicates treatment efforts and reduces survival rates. The aggressive behavior of these tumors and their tendency to metastasize make the early detection, through familial (germline) genetic evaluation, crucial to improve prognosis.^23^

## Diagnostic Workup in TNENs

### Biochemical Assessment

**Figure 1:**
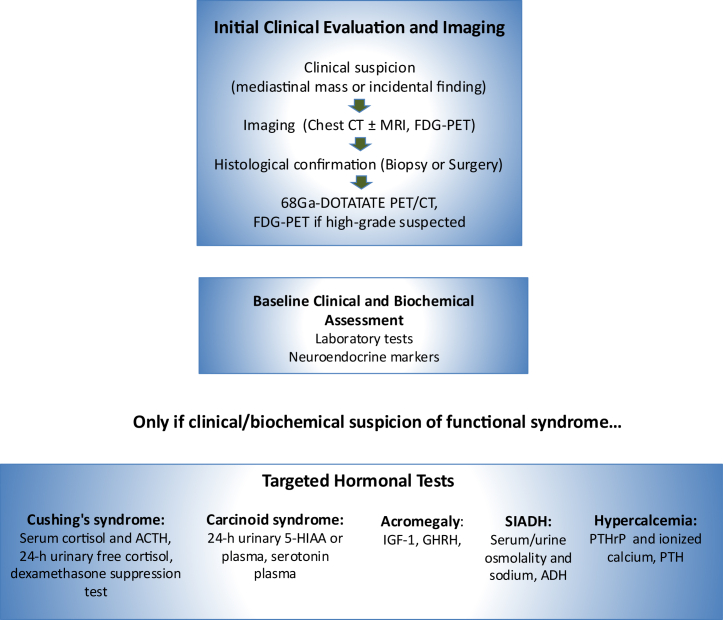
Clinical diagnostic workup algorithm for TNENs. ACTH, adrenocorticotropic hormone; ADH, antidiuretic hormone; CT, computed tomography; FDG-PET, fluorodeoxyglucose positron emission tomography; GHRH, growth hormone–releasing hormone; IGF-1, insulin-like growth factor 1; MRI, magnetic resonance imaging; PTH, parathyroid hormone; PTHrP, parathyroid hormone–related peptide; SSTR, somatostatin receptor; 5-HIAA, 5-hydroxyindoleacetic acid.

A baseline laboratory panel, including a complete blood cell count, renal and liver profile, electrolytes, and serum calcium, is recommended for every newly suspected TNEN, along with broad neuroendocrine markers such as chromogranin A and neuron-specific enolase, which are elevated in a significant proportion of thoracic NETs (Fig. 1).^7^

When clinical clues suggest a functional tumor, targeted tests confirm ectopic hormone production: serum cortisol and ACTH for suspected Cushing syndrome^9^^,^^10^; 24-hour urinary 5-HIAA or plasma serotonin for carcinoid syndrome^15^; growth hormone-releasing hormone and IGF-1 for acromegaly^19^; serum/urine osmolarity and sodium with ADH for SIADH^21^; and PTHrP with ionized calcium (occasionally PTH) for PTHrP-mediated hypercalcemia.^22^ Documenting these abnormalities not only confirms the diagnosis of paraneoplastic syndromes but also guides perioperative management, allows biochemical follow-up after resection, and helps tailor systemic therapy when hormonal control is required.

### Diagnostic Imaging

Imaging studies represent the cornerstone of the initial evaluation of suspected TNENs. Chest radiographs are often the first imaging modality in patients with suspected mediastinal masses. Although it detects the presence of a mediastinal mass, its sensitivity and specificity are limited. CT scans are the mainstays of imaging for TNENs, providing detailed anatomical information. CT scans offer excellent spatial resolution, allowing for discerning location, morphology, shape, margins, size, density, enhancement, and relationship to, or invasion of, adjacent structures.^24^ Overall, CT is equal or superior to MRI in evaluating mediastinal masses except for cysts or cystic components of tumors.^25^ Although MRI is not usually used to assess thymic tumors, it can help to distinguish solid from cystic lesions, evaluate cystic or necrotic components of a mass, and identify enhancing septage within cystic lesions and areas of subtle local invasion.^26^ FDG-PET is particularly helpful in high-grade TNENs, such as LCNEC and SCNEC, which typically reveal high FDG avidity, whereas well-differentiated tumors may have limited uptake.^27^ Despite the limited literature on this topic, FDG-PET may help identify areas of high metabolic activity, particularly in highly aggressive or high-proliferation TNENs, including proposed NET G3.

Gallium-68 DOTATATE PET/CT has emerged as a highly sensitive and specific imaging modality for evaluating NENs, including those of the thymus.^28^ In clinical practice, a Gallium-68 DOTATATE PET/CT should be performed after histologic confirmation of TNEN (or when a well-differentiated NET is highly suspected).^29^ In these patients, Gallium-68 DOTATATE PET/CT offers several advantages. First, it allows for the precise localization and characterization of the primary tumor, thereby facilitating the delineation of its extent and relationship with surrounding structures, which is particularly relevant given the proximity of thymic tumors to vital mediastinal structures. Second, the high sensitivity helps identify lymph node involvement and even small distant metastases, which is critical for accurate staging and treatment planning at the time of diagnosis,^30^^,^^31^ and is found to be useful in monitoring treatment response and detecting disease recurrence.^32^ In addition, Gallium-68 DOTATATE PET/CT plays a pivotal role in therapeutic decision-making by selecting patients with sufficient somatostatin receptor expression to be suitable candidates for PRRT^33^

## Surgical Management

Complete, margin-negative (R0) surgical resection is the primary treatment for TNETs, and it is the strongest independent prognostic factor for OS, as demonstrated in large real-world cohorts and multicenter studies.^34^^,^^35^ In addition, including systematic lymph node dissection is advisable due to the high incidence of nodal metastasis and its impact on staging and prognosis.^36^

The International Thymic Malignancy Interest Group (ITMIG) recommends that lymph node dissection in stage II or higher thymic malignancies should include the right paratracheal (RPN, N2) station when indicated, as this is a key route of spread. Accordingly, the North American Neuroendocrine Tumor Society (NANETS) also emphasizes the importance of complete mediastinal lymphadenectomy for optimal staging and disease control.^37^^,^^38^

For resectable TNETs, the standard surgical approach is total or extended thymectomy with en bloc resection of involved mediastinal fat and adjacent structures. Median sternotomy is preferred for suspected invasion of the lung, pericardium, or great vessels, whereas minimally invasive approaches (video-assisted or robotic thymectomy) are appropriate for small, well-localized tumors in experienced centers, provided R0 margins and adequate nodal assessment are achieved.

Postoperative radiotherapy should be considered, especially for Masaoka–Koga stage III to IV and IIB TNETs, as Surveillance, Epidemiology, and End Results database analyses reveal a significant survival benefit in these groups.^39^

## Treatment Options

The treatment of TNENs is complex and relies on the tumor's histologic subtype, stage, and the patient's overall health condition. A multidisciplinary approach involving thoracic surgeons, oncologists, radiologists, and pathologists is crucial for the optimal management of these cases. Surgical resection, when feasible, remains the cornerstone of treatment for TNENs, as achieving complete resection has been found to significantly improve survival outcomes.^39^

Retrospective studies have revealed no clear evidence of benefit of adjuvant treatment of TNETs.^40^ Currently, adjuvant therapy is not recommended by the American (NCCN) and European (ENETS and ESMO)^1^^,^^41^ guidelines following R0 resection of typical or atypical thymic carcinoids.

In contrast, adjuvant therapies are often necessary for high-grade LCNECs and SCNECs due to their elevated risk of recurrence, typically a combination of etoposide with platinum-based chemotherapy. Radiotherapy is a standard adjuvant treatment following surgical resection, particularly for high-grade TNENs and cases with incomplete resection (R1 or R2 resection). Postoperative radiotherapy should be considered, especially in Masaoka–Koga stage III to IV and IIB TNETs, as Surveillance, Epidemiology, and End Results database analyses indicate a significant survival benefit in these groups.

It may also be used as a primary modality for unresectable tumors or as palliative therapy for alleviating symptoms.^39^

In advanced TNENs, particularly TNECs, platinum-based chemotherapy (cisplatin or carboplatin with etoposide) remains the first-line treatment. This strategy reflects the approach used for high-grade pulmonary neuroendocrine carcinomas, considering their similar biological aggressiveness and the absence of disease-specific prospective trials.

A recent retrospective study evaluated the effectiveness of combining etoposide and platinum-based chemotherapy in treating only advanced and predominantly high-grade TNENs. The study included 16 patients with advanced TNENs, and reported a 31.3% objective response rate and 81.3% disease control rate (DCR), with a median PFS of 7.2 months and median OS of 50.4 months. Grades 3 to 4 hematologic toxicity occurred in half the patients, with no treatment-related deaths.^42^

In advanced, well-differentiated cases of TNETs where surgery is not feasible, SSAs (octreotide, lanreotide) are used as a first-line treatment option given their antiproliferative and antiangiogenic effects, as well as their utility in managing potentially associated carcinoid symptoms.^4^

The treatment strategy for advanced TNETs is primarily derived from pivotal trials conducted in GEP-NETs and lung carcinoids. The extreme rarity of these tumors presents significant challenges in obtaining subtype-specific data and conducting focused research studies. For instance, although the PROMID and the CLARINET studies both demonstrated the antiproliferative effect of octreotide LAR in well-differentiated metastatic mid-gut NETs (PROMID) and grades 1 to 2, nonfunctioning, stable enteropancreatic NETs (CLARINET), TNETs were either absent or not reported separately, with anecdotal patients categorized as ‘other’ primary sites.^43^^,^^44^

However, retrospective real-world data support the role of SSAs in all patients who demonstrate SSTR expression. In a large retrospective study conducted by French networks, which included 74 patients diagnosed with advanced well-differentiated TNETs (TNECs excluded), 46% of patients received SSAs, primarily in earlier lines of treatment and in tumors exhibiting indolent behavior. In this study, somatostatin receptor expression was demonstrated in 74% of assessable cases, and a DCR exceeding 60% was observed, thus supporting the role of SSAs as a viable therapeutic option for selected patients in the real-world setting.^4^ Chemotherapy was administered in 68% of cases and used as first-line treatment in 41%. The most frequently employed regimens were platinum–etoposide (28%) and 5-FU–oxaliplatin (32%), with observed objective response rates of 38% and 29%, respectively. Although cytotoxic chemotherapy has traditionally been reserved for poorly differentiated neuroendocrine carcinomas, these real-world data support its relevance also in well-differentiated TNETs, further consistent with NCCN recommendations.^45^ Importantly, expert pathologists in the study centrally reviewed all cases and classified them according to the 2021 WHO criteria. NET G3, defined as well-differentiated tumors with a Ki-67 index more than 20%, accounted for 31% of the cohort, and among 74 patients of the overall cohort, 57% received chemotherapy and yielded 38% of ORR, with platinum-based combinations.^4^

These findings emphasize the potential benefits of chemotherapy in high-burden or symptomatic cases of newly identified but not formally recognized NET G3, where heightened proliferative activity may warrant a more aggressive approach. However, more robust data are needed to define optimal treatment strategies for TNETs once this emerging category is formally recognized.

The mTOR inhibitor everolimus has demonstrated efficacy in lung carcinoids within the phase II RADIANT-2 trial^46^ and the phase III RADIANT-4 study.^47^ However, only one patient with TNETs was included. The phase II LUNA trial enrolled 124 patients with well-differentiated, advanced lung [116/124, (93.5%)] or TNETs [8/124, (6.5%)]. Furthermore, 41 received pasireotide, 42 received everolimus, and 41 received the combination regimen. At 9 months, the proportion of patients who remained progression-free was 39.0%, 33.3%, and 58.5% in the pasireotide, everolimus, and combination arms, respectively, but no subanalyses of efficacy by tumor site (lung versus thymus) were conducted.^48^

A small study involving four patients with recurrent TNETs, two with atypical carcinoids having necrosis, and two with large cell features reported a mean duration of disease stabilization of 20.8 months under everolimus, with better outcomes in cases with lower Ki-67 indices.^49^

One additional French retrospective study included 29 individuals with TNENs (39%) who received everolimus, achieving a DCR of 58.6% and a median progression-free survival (PFS) of 6.5 months. The median number of systemic treatment lines received was 3 (range: 1–8). Everolimus was among the most frequent systemic options. These real-world data support its role in selected patients, particularly those with indolent or slowly progressive disease.^4^

Despite the limited number of patients with TNET reported in the literature and the extrapolation of most data from pulmonary or GEP-NETs, current international guidelines (e.g., ESMO, NCCN) consider everolimus a first-line treatment option in atypical carcinoids or a second-line option following progression on somatostatin analogues in TNETs (off-label for thymic primaries in the United States/European Union).

Temozolomide has emerged as a potential therapeutic option for treating TNENs, particularly for low-proliferative tumors. A retrospective study of 36 patients, including seven with thymic carcinoids, demonstrated that temozolomide achieved a median time to progression of 7 months. Among these patients, 14% experienced partial responses, whereas 53% maintained stable disease.^50^

Additional evidence from a retrospective study conducted at a single tertiary referral center in Sweden included 28 patients with TNENs, stratifying them by proliferation index (<10% versus ≥10%). Temozolomide monotherapy achieved a median time to progression of 20.5 months and a 20% partial response rate, whereas platinum-based regimens resulted in a median time to progression of 18 months and a higher partial response rate of 38%.^51^ Notably, it is also reported that patients with low-proliferation tumors (Ki67 < 10%) demonstrated markedly longer median survival compared with those with higher proliferation indices, suggesting the prognostic relevance of Ki67 in this setting.

Temozolomide, as monotherapy or in combination with capecitabine, is recommended by international guidelines such as ESMO and NCCN as a therapeutic option for atypical carcinoids and extrapulmonary NETs, including thymic primaries. Although its use in TNETs remains off-label in both the United States and European Union, it is frequently reimbursed in the United States and is often accessible in Europe through national off-label prescribing frameworks, particularly in referral centers.^1^

The tyrosine kinase inhibitor (TKI) sunitinib has also demonstrated anecdotal activity. In one reported case, a patient with typical thymic carcinoid received sunitinib in combination with a somatostatin analogue as neoadjuvant therapy, leading to measurable tumor shrinkage within 3 weeks.^52^ Although this result is promising, further studies are necessary to determine the efficacy and potential positioning of sunitinib in the treatment of TNETs.

Despite the increasing use of immune checkpoint inhibitors (ICIs) in TETs, most major immunotherapy trials have not specifically reported outcomes for TNECs. One study reported the inclusion of six patients (15%) with neuroendocrine histology among 39 cases of advanced thymic carcinoma, with a median OS of 24.9 months and a median PFS of 4.2 months, and no histology-specific subanalysis.^53^ Beyond that, case-level evidence suggests limited efficacy of ICIs in TNENs. In a recent retrospective Chinese series involving 51 patients with TNENs, two patients with atypical carcinoids achieved durable partial responses to toripalimab. In contrast, one patient with LCNEC did not respond, highlighting histologic heterogeneity in immunotherapy sensitivity.^54^ These findings, although taken cautiously given the small numbers, highlight a potential differential sensitivity to immunotherapy based on histologic subtype and warrant further investigation in larger, subtype-specific cohorts.

PRRT with ^177^Lu-DOTATATE has emerged as a potential therapeutic option for patients with advanced SSTR-positive NETs. Although TNETs were not included in the pivotal NETTER-1 trial, increasing real-world evidence supports the use of radioligand therapy in this rare subgroup.^55^

Again, in the French retrospective study, PRRT was administered to four patients with advanced TNET, all with positive somatostatin receptor imaging. Despite appropriate selection, disease progression occurred in 75% of cases, possibly due to low or heterogeneous receptor expression, bulky disease, or bone metastases, highlighting the need to collect more prospective data to clarify the role of PRRT in this setting.^4^

Case reports and institutional experiences support PRRT as a treatment option. Halperin et al.^56^ described a patient with metastatic thymic malignancies who experienced a substantial partial response after four cycles of PRRT, with marked tumor shrinkage in the pleura, liver, and heart. Another case report detailed an 8-year survival in a patient with metastatic thymic carcinoid treated with multiple PRRT cycles, achieving long-term disease control.^57^

TNETs typically demonstrate strong uptake on ^68^Ga-DOTATATE PET/CT, and patients with low to intermediate Ki-67 indices appear more likely to benefit from PRRT. Toxicity is generally manageable and consistent with that observed in GEP-NETs, though long-term renal and hematologic monitoring is advised.

Although prospective trials evaluating PRRT in TNENs are currently lacking, the ESMO and NCCN guidelines acknowledge PRRT as a therapeutic option in this setting.^1^ Specifically, PRRT is recommended for patients with somatostatin receptor–positive tumors who progress on somatostatin analogues. It may also be considered as an alternative to cytotoxic chemotherapy in selected patients, particularly those with refractory carcinoid syndrome and homogeneously positive somatostatin receptor imaging (SRI) across all Response Evaluation Criteria in Solid Tumors–assessable lesions. In rare cases, PRRT may even be used as a first-line therapy in SRI-positive patients presenting with bulky or rapidly progressing disease despite medical therapy. Given its technical complexity and potential toxicity, PRRT should ideally be delivered in specialized centers with expertise in nuclear medicine and multidisciplinary management of neuroendocrine neoplasms. Importantly, PRRT remains off-label for TNENs in both the United States and European Union, and its use should be restricted to specialized centers due to its technical complexity and potential toxicity.

**Figure 2:**
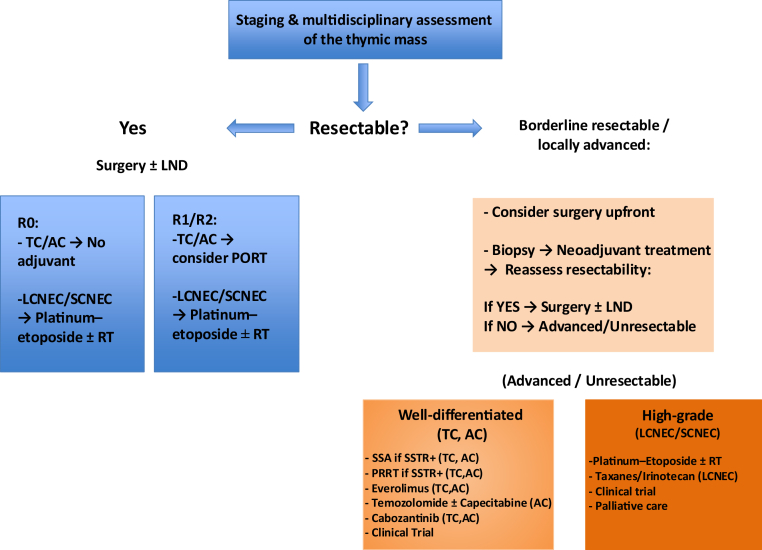
Treatment algorithm for thymic neuroendocrine tumors (TNENs). AC, atypical carcinoid; Chemo, chemotherapy; LCNEC, large-cell neuroendocrine carcinoma; LND, lymph node dissection; NET G3, well-differentiated neuroendocrine tumor, grade 3; PRRT, peptide receptor radionuclide therapy; RT, radiotherapy; SSA, somatostatin analog; SCNEC, small-cell neuroendocrine carcinoma; SSTR, somatostatin receptor; TC, typical carcinoid; TNEN, thymic neuroendocrine neoplasm.

Finally, cabozantinib, a multi-target TKI, was recently evaluated in the pivotal CABINET trial (NCT03375320), a randomized phase III study that enrolled patients with progressive, well- or moderately differentiated pancreatic and extra-pancreatic NETs who had received at least one prior therapy. Notably, TNETs were explicitly included in the extra-pancreatic cohort where cabozantinib significantly improved mPFS compared with placebo (8.5 versus 4.2 mo; hazard ratio 0.40; *p* < 0.0001). Although objective response rates remained modest (5%), disease stabilization was clinically meaningful.^58^ These findings led to FDA approval of cabozantinib in February 2025 for the treatment of advanced well-differentiated pancreatic and extra-pancreatic NETs, including thymic primaries. This has recently been incorporated into the NCCN guidelines with a category 1 recommendation for advanced extra-pancreatic NETs, supporting its potential utility in progressive TNETs. Although subgroup analyses specific to TNETs were not reported, the inclusion criteria support its potential role in this rare setting, particularly in patients progressing on somatostatin analogues or everolimus. All available therapies are found in Figure 2 and are detailed in Table 2.

**Table 2:** Therapeutic Overview of Thymic Neuroendocrine Tumors

Subtype	Stage	Surgery	Adjuvant Therapy	Medical Therapy (Advanced Disease)	Comments
Typical carcinoid	Localized	Recommended when feasible, Locoregional therapies	Not routinely recommended		Often diagnosed late; long-term follow-up required
	Advanced	Rarely feasible	—	Observation (if asymptomatic); SSAs if SSTR+; PRRT^a^ if SSTR+; Everolimus^b^ in progressive disease; Cabozantinib^c^	Indolent but prone to recurrence
Atypical carcinoid	Localized	Strongly recommended, Locoregional therapies	Consider in high-risk cases		Intermediate behavior; requires multidisciplinary discussion
	Advanced	Rare	—	SSAs; Everolimus^b^; Temozolomide-d ± Capecitabine; PRRT^a^ if SSTR+; (Cabozantinib^c^); Platinum-based chemotherapy	May benefit from combined treatments
LCNEC	Localized	Recommended if resectable	Yes (platinum–etoposide ± radiotherapy depending on resection status)		High-grade; parallels thymic carcinoma approach
	Advanced	Rare	—	Platinum–etoposide; consider taxanes or irinotecan; limited role for PRRT^a^ due to low SSTR expression; supportive care in poor PS	Aggressive course; early systemic therapy essential
SCNEC	Localized	Recommended if feasible	Yes (platinum–etoposide ± radiotherapy)		Extremely aggressive; extrapolated from SCLC protocols
	Advanced	Rare	—	Platinum–etoposide; limited evidence for targeted therapy or PRRT^a^; consider enrollment in clinical trials	Early dissemination common; prognosis poor

*Note*: Summary of recommended surgery/locoregional management, adjuvant use, and systemic options for advanced disease.

ESMO, European Society for Medical Oncology; EU, European Union; FDA, Food and Drug Administration; NCCN, National Comprehensive Cancer Network; US, United States.

a PRRT is currently off-label for TNETs in the US and EU. Its use may be considered in patients with somatostatin receptor–positive tumors progressing after somatostatin analogues, but only in specialized centers with expertise in nuclear medicine and multidisciplinary neuroendocrine tumor management.

b Everolimus is also off-label for TNETs in the US and EU. Evidence supporting its use is extrapolated from trials in lung and GEP-NETs, with limited direct data in thymic primaries.

c Cabozantinib is FDA approved (March 2025) for advanced, progressive well-differentiated extra-pancreatic NETs, including thymic primaries. However, it remains off-label in the EU, and subgroup efficacy data in TNETs are not yet available.

d Temozolomide is not formally approved for TNETs and remains an off-label option in both the US and EU. However, it is recommended by international guidelines such as ESMO and NCCN for atypical carcinoids and extrapulmonary NETs, including thymic primaries. In the US, reimbursement is often granted when supported by guideline-based justification, but in Europe, access is determined by national off-label use policies and often permitted in specialized settings.

## Ongoing Studies

Despite their extreme rarity, TNENs are now included in several ongoing clinical trials exploring new therapeutic strategies; however, no trials are enrolling only TNENs.

The LEVEL trial (NCT05918302) is a phase III, randomized, international study evaluating ^177^Lu-edotreotide (a DOTA-TOC analogue structurally related to ^177^Lu-DOTATATE and characterized by high affinity for SSTR2 with additional binding to SSTR5) versus everolimus in patients with well- or moderately differentiated lung and TNETs (TC and AC), whether treatment naive or previously treated. With a target enrollment of 120 patients, the trial aims to assess PFS as its primary end point and is expected to report in 2028.^59^

Another study, NCT06121271, is a phase II trial investigating the off-label use of ^177^Lu-DOTATATE in patients with bronchial and TNENs. Although not yet recruiting, this open-label, single-arm trial aims to enroll 75 to 110 participants to prospectively evaluate the safety and efficacy of PRRT in unresectable or metastatic SSTR-positive bronchial and thymus primaries in a 2-year follow-up period (phase II study; NCT06121271).^60^

The NCT03070301 is a phase II study evaluating the efficacy of the CDK4/6 inhibitor ribociclib in combination with everolimus in patients with well- or moderately differentiated foregut NETs, including thymic primaries. This approach is supported by the frequent activation of the cyclin D-CDK4/6-retinoblastoma pathway in foregut NETs, which drives cell-cycle progression, together with constitutive activation of the PI3K/AKT/mTOR axis. Preliminary results revealed a DCR above 60%, although hematologic toxicity limited broader applicability.^61^

So far, no clinical data are available on bispecific T-cell engagers in TETs. Encouraging signals, however, have been observed in other NENs. At ASCO 2025, Capdevila et al.^62^ presented phase I data with tarlatamab in pulmonary and extrapulmonary neuroendocrine carcinomas, and Patel et al.^63^ reported clinically relevant activity in pulmonary LCNEC. Along with the DeLLphi program in SCLC, these findings provide a rationale to explore bispecifics, such as tarlatamab, in thymic malignancies.

Collectively, these trials underscore the growing commitment and clinical interest in developing dedicated therapeutic strategies for TNETs, offering new prospects for improved survival outcomes in this rare subgroup.

## Conclusion

TNETs represent a rare and heterogeneous subset of thoracic malignancies, ranging from indolent typical carcinoids to highly aggressive neuroendocrine carcinomas. Due to their low incidence and underrepresentation in clinical trials, current treatment approaches are often derived from strategies used for lung carcinoids and GEP-NETs. This results in significant uncertainties regarding optimal therapeutic sequencing, prognostic stratification, and the role of newer agents in the TNET landscape.

Although cytotoxic chemotherapy, particularly platinum–etoposide, remains the backbone for aggressive and high-grade TNECs, retrospective real-world data suggest that even well-differentiated TNETs may respond to chemotherapy. These findings emphasize the clinical importance of accurately identifying NET G3 tumors, as increased proliferative activity may require a more aggressive treatment approach even though the morphology is well differentiated.

However, optimal management remains uncertain, with evidence largely limited to small retrospective series, and warrants further investigation.

Among emerging treatment strategies, the recent FDA approval of cabozantinib for extra-pancreatic NETs, including thymic primaries, marks an important milestone and expands the poor therapeutic options for these patients. PRRT has gained increasing attention as a rational and biologically targeted option in patients with somatostatin receptor–positive tumors. Although prospective data specific to TNETs are lacking, retrospective analyses and case series have reported encouraging DCRs and prolonged PFS, particularly in slowly progressive disease. Furthermore, ongoing prospective trials, such as the LEVEL study, are expected to provide definitive data on the efficacy of PRRT in the thymic setting. Given its favorable toxicity profile and mechanism of action, PRRT is increasingly being considered not only as a salvage therapy but potentially as a first-line option in selected patients, especially those with refractory carcinoid syndrome, bulky disease, or diffuse SRI-positive uptake, as NETTER-2 revealed for GEP-NETs. However, its use remains off-label and its administration should be restricted to specialized centers with high expertise in nuclear medicine and multidisciplinary NEN management.

In conclusion, the management of TNETs requires a multidisciplinary approach that considers histologic grade, functional status, receptor expression, and tumor behavior. Although treatment options are expanding, robust prospective data remain limited, and collaborative efforts are urgently needed to develop dedicated clinical trials, establish histology-specific guidelines, and incorporate molecular profiling into clinical practice. The inclusion of TNETs in basket trials and international registries will be crucial to improving outcomes in this rare and challenging disease.

## CRediT Authorship Contribution Statement

**Erica Pietroluongo**: Conceptualization, Data curation, Formal analysis and interpretation of data, Writing – original draft, Final approval of the manuscript.

**Christine M. Bestvina**: Writing – review and editing, Final approval of the manuscript.

**Rachel Brattin:** Data curation, Final approval of the manuscript.

**Pietro De Placido**: Writing – review and editing, Final approval of the manuscript.

**Anna Di Lello:** Data curation, Final approval of the manuscript.

**Waqas Haque:** Data curation, Final approval of the manuscript.

**Alessandra Esposito**: Data curation, Final approval of the manuscript.

**Roberto Bianco**: Writing – review and editing, Final approval of the manuscript.

**Noura Choudhury**: Writing – review and editing, Final approval of the manuscript.

**Marina Chiara Garassino**: Conceptualization, Writing – review and editing, Supervision, Final approval of the manuscript.

## Disclosure

Dr. Garassino reports having advisory or consulting roles for Bristol Myers Squibb, Merck Sharp & Dohme, AstraZeneca, Novartis, Takeda, Roche, Sanofi, Celgene, Daiichi Sankyo, Pfizer, Seagen, Lilly, GlaxoSmithKline, Bayer, Blueprint Medicines, Janssen, Regeneron, AbbVie, Mirati, Merck, Boehringer Ingelheim, Abion, Gilead, IO Biotech, Novocure, Janssen Oncology, and Revolution Medicines; receiving speaker honoraria from MSD Oncology, AstraZeneca/MedImmune, GlaxoSmithKline, Takeda, Roche, Bristol Myers Squibb, Daiichi Sankyo/AstraZeneca, Regeneron, Pfizer, Blueprint Medicines, Novartis, Sanofi/Aventis, Medscape, Oncohost, and Revolution Medicines; receiving research grants to the institution from Bristol Myers Squibb, Merck Sharp & Dohme, Roche/Genentech, AstraZeneca/MedImmune, AstraZeneca, Pfizer, GlaxoSmithKline, Novartis, Merck, Incyte, Takeda, Spectrum Pharmaceuticals, Blueprint Medicines, Lilly, Ipsen, Janssen, Exelixis, MedImmune, Sanofi, and Amgen; and receiving travel support from Pfizer, Roche, AstraZeneca, and Merck. All relationships are outside the submitted work. Dr. Bianco reports having advisory or consulting roles for Bristol Myers Squibb, Merck Sharp & Dohme, Pfizer, AstraZeneca, Lilly, and Novartis. All relationships are outside the submitted work. Dr. Choudhury reports receiving institutional research funding from Amgen, AbbVie, Monte Rosa Therapeutics, Roche/Genentech, Merck, Harpoon Therapeutics, Janux Therapeutics, Seagen/Pfizer, and DualityBio; having consulting or advisory roles for AbbVie, Merck, Harpoon Therapeutics, Eli Lilly, and Amgen; receiving honoraria from MJH Life Sciences and IDEOlogy Health; and receiving royalties from Wolters Kluwer. All relationships are outside the submitted work. Dr. Bestvina reports receiving research grants to the institution from AstraZeneca and Bristol Myers Squibb; having advisory or consulting roles for AbbVie, Amgen, AstraZeneca, Bristol Myers Squibb, Catalyst, Daiichi Sankyo, EMD Serono, Genentech, Gilead, Guardant, Jazz, Johnson & Johnson, Lilly, Mirati, Novartis, Pfizer, Tempus, and Turning Point Therapeutics; and receiving travel support from AstraZeneca, Bristol Myers Squibb, and Guardant. All relationships are outside the submitted work. Dr. Pietroluongo reports being supported by a scholarship from Associazione TUTOR (Rare Thoracic Cancer Patients Association) for the academic years 2024 to 2025. All relationships are outside the submitted work. The remaining authors declare no conflict of interest.
