# Supplementary material for: Thymic Neuroendocrine Tumors: Evolving Insights and Innovative Approaches
Source: JTO Clin Res Rep. 2025 Nov 20;7(2):100935. doi: 10.1016/j.jtocrr.2025.100935 (PMC12828808; doi:10.1016/j.jtocrr.2025.100935)
Supplement: Supplementary Table 1 [file mmc1.docx]

| **Setting** | **Regimen / Modality** | **Study (type)** | **N (TNEN)** | **ORR** | **Disease control** | **Median PFS (mo)** | **Median OS** |
| --- | --- | --- | --- | --- | --- | --- | --- |
| Neoadjuvant | Mixed chemo (platinum-based in most) | Corsini 2019, MD Anderson (retrospective single-center) | 16 | 25%  (4/16) | 9 SD /16; 3 indet. | NR | NR |
| Neoadjuvant | Various: chemo (n=6), SSA (n=4), CRT (n=1) | Chen 2022, Orphanet J Rare Dis (retrospective single-institution) | 11 | NR | NR | NR | 61.6 mo |
| Neoadjuvant (case) | Sunitinib + octreotide | Dham 2008, J Thorac Oncol (case report) | 1 | PR | PR | NR | NR |
| Neoadjuvant (case) | Cytotoxic chemotherapy (atypical carcinoid) | Ventura 2017, J Thorac Dis (case report) | 1 | PR | PR | NR | NR |
| Neoadjuvant | Induction therapy (CT/RT) | **Filosso 2015,** ITMIG/ESTS (database) | 25 out of 205 | **NR** | NR | NR | 5-yr **OS 68%** (entire cohort) |

**Supplementary Table 1: Neoadjuvant Approach in Thymic Neuroendocrine Tumors**

Overview of published series and case reports of neoadjuvant therapy, including the treatments used and main reported outcomes.

**Abbreviations:** TNEN, thymic neuroendocrine tumor; ORR, objective response rate; PFS, progression-free survival; OS, overall survival; SSA, somatostatin analog; CRT, chemoradiotherapy; ITMIG, International Thymic Malignancy Interest Group; ESTS, European Society of Thoracic Surgeons; NR, not reported; SD, stable disease; PR, partial response; CT, chemotherapy; RT, radiotherapy; yr, years; mo, months.
